# Factors Associated With the Utilisation and Unmet Need for Modern Contraceptives Among Urban Women in Kenya: A Cross-Sectional Study

**DOI:** 10.3389/fgwh.2021.669760

**Published:** 2021-12-16

**Authors:** Catherine Akoth, James Odhiambo Oguta, O'Brien M. Kyololo, Martin Nyamu, Michael Ndung'u Ndirangu, Samwel Maina Gatimu

**Affiliations:** ^1^Institute of Tropical and Infectious Diseases, Faculty of Health Sciences, University of Nairobi, Nairobi, Kenya; ^2^School of Health and Related Research, University of Sheffield, Sheffield, United Kingdom; ^3^Health Section, UNICEF, Eastern and Southern Africa Regional Office, Nairobi, Kenya; ^4^School of Nursing, Moi University, Eldoret, Kenya; ^5^School of Economics, University of Nairobi, Nairobi, Kenya; ^6^Diabetic Foot Foundation of Kenya, Nairobi, Kenya

**Keywords:** modern contraceptive use, family planning, unmet need, PMA survey, Kenya, urban

## Abstract

**Background:** Family planning (FP) is a key intervention in improving maternal and child health. Hence, we assessed the factors associated with utilisation and unmet need for modern contraceptives among urban women in Kenya.

**Methods:** The study used pooled data on 10,474 women 15–49 years from the seven rounds of the performance monitoring for accountability surveys collected between 2014 and 2018. The surveys were conducted in 11 of the 47 counties of Kenya using a multistage cluster design. Sample characteristics were described using frequencies and percentages while factors associated with utilisation and unmet need for modern contraceptives were assessed using multivariable logistic regressions.

**Results:** The prevalence of modern contraceptives use and unmet need for FP among urban women in Kenya was 53.7% [95% confidence interval (CI) 52.1–55.3%] and 16.9% (15.8–18.1%), respectively. The use of modern contraceptive was associated with the county of residence, age, marital status, parity, education, household wealth quintile, exposure to media, and survey year. Teenagers, poorest urban women, women with no formal or primary level of education and those who seek services at a dispensary or health centres had higher odds of unmet need for FP while women who resided in Kitui and Nyamira counties had reduced odds of unmet need for FP. The odds of unmet need decreased with the survey year while that of modern contraceptive use had an inverse trend.

**Conclusion:** Overall modern contraceptive use in urban areas is lower than the national average while the unmet need for FP is higher than national average, highlighting a potential urban-rural disparity in FP indicators in Kenya. Individual sociodemographic and socioeconomic and contextual factors are associated with the use of modern contraceptive and unmet need for FP among urban women in Kenya. Urban family planning policies and programmes in Kenya need to focus on strengthening urban healthcare systems to provide equal and accessible FP services, especially targeted towards teenagers and young women and those of low socioeconomic status.

## Background

Birth spacing and limiting using modern family planning (FP) methods have the potential to avert 1.5 million maternal deaths and about two million infant deaths annually and contribute to the overall economic growth and development (1, 2). Unfulfilled demand for FP, on the other hand, contributes to unplanned pregnancies and unsafe abortions (3, 4). Despite these findings, the availability and access to modern FP among women of reproductive age (15–49 years) remains a challenge globally and particularly in low- and middle-income countries (LMICs) (1). For instance, while 63% of women of reproductive age globally use some form of contraception, 11.5% have an unmet need for FP (5), with only about one in three women using contraception and 23.4% having an unmet need for FP in sub-Saharan Africa (1, 6).

In Kenya, remarkable progress towards attaining universal access to FP has been made with an increase in modern contraceptive prevalence rate (CPR) from 53.2% in 2014 (7) to 58% in 2020 (8); but still about one in ten women have an unmet need for FP (9). However, successive national demographic and health surveys have continued to show an urban-rural disparity in contraceptive use (7, 10). For example, in 2014, about 40% of urban women were not using modern contraceptives and a further 13% had an unmet need for FP (7, 10). Recent studies have also found a persistent low use of modern contraceptive and a high unmet need in urban areas (11, 12). This is despite, a general expectation that access to FP services is better in urban than rural areas hence a need to explore this phenomenon.

Furthermore, Kenya like most sub-Saharan Africa countries is experiencing rapid urbanisation; with the urban population growing from 7.8% in 1962 (13) to 31.1% in 2019 (14) and is projected to reach 45.7% in 2050 (15). This rapid urban population growth is attributable in part to rural to urban migration and majorly to natural population growth (16), reinforcing the need for improved FP services in urban areas. Unfortunately, inadequate infrastructure and housing in urban areas have resulted in 56% of the urban population living in slums and other informal settlements (17); 36% of whom live in Nairobi county (18). These informal urban settlements are often characterised by overcrowding, poor access to healthcare including reproductive health services (19) and poor sexual and reproductive health outcomes (20). Besides, only 73% of health facilities in urban areas in Kenya offer FP services compared to 91% in rural areas; and even fewer facilities provide specific FP methods and only 44% offering FP services for adolescents (21).

Although the urban fertility rate has been declining with an overall increase in modern contraceptive use, the unmet need for contraception is still high and greatest among the urban poor (9). Therefore, urbanisation, urban population dynamics and the rise of urban informal settlements cannot be ignored in the bid to achieve health goals including FP. Consistent FP programmes to build on gains of previous urban FP programs such as *Tupange* (22) are needed to address the increased demand and need for FP in urban areas but to also address other confounders of FP use including poverty, low education level, exposure to FP messages in the media and FP commodities' stockouts (11, 12). Also, while previous studies have consistently shown the prevalence of unmet need for FP to be lower in urban areas than in rural areas (11, 12), a possible rich-poor gap in unmet need for FP among urban women may be masked. Hence, to better inform urban FP programming and policies in Kenya, the study aimed to determine factors associated with modern contraceptive use and unmet needs for FP among urban women in 11 counties in Kenya.

## Methods

### Study Setting

Kenya is a lower-middle-income country with a fertility rate of 3.6 births per woman (23) and a population of about 47.6 million−30.1% of whom lives in urban areas (14). It has a gross domestic product of US$ 1816.6 per capita, human development index of 0.6 and a gender inequality index of 0.5 (24). Health care services are provided through the six levels of care from community to national referral services. The 47 subnational (county) governments oversee health service delivery at the country level while the national government oversees health policy formulation, training, and management of national referral hospitals. Family planning services are provided at all levels of health care but only about 85% of health facilities currently provide FP services with 97 and 79% of government and private facilities, and 89–90 and 99% of dispensaries and health centres and public primary hospital providing the services, respectively (21).

### Data Source

The study used pooled data from seven rounds of the performance monitoring for accountability (PMA) surveys (25–31). The PMA is a multi-country survey on the sexual and reproductive health of women of reproductive age (32). In Kenya, the survey has been conducted since 2014 in 11 counties (*Kericho, Kiambu, Nandi, Siaya, West Pokot, Bungoma, Kitui, Kilifi, Nyamira, and Nairobi*) using a multistage cluster design, with the counties as the strata. The included households were systematically selected from enumeration areas that were randomly selected from the Kenya National Bureau of Statistics (KNBS) master frame. In the sampled households, all females aged 15–49 years and who consented to the study were interviewed. The survey also included randomly selected service delivery point (SDP) offering FP services in the selected communities. The management staff in the sampled health facilities were interviewed on behalf of the facility. The survey methodology for PMA surveys has been described further elsewhere (25–31).

This study included data from women aged 15–49 years and from urban health facilities. A total of 35,792 women of reproductive age were interviewed; 13,154 of whom were from urban areas. Of the 13,154 urban women, 2,680 missing observations were excluded resulting to a final sample of 10,474 women for analysis for modern contraceptive use. The analysis for unmet need for FP included 8,722 women after excluding 4,432 women (3,512 who were not sexually active, 577 infecund and 343 with missing observations).

### Study Variables

Modern contraceptive use was defined as “*the use of a product or medical procedure that interferes with reproduction from acts of sexual intercourse*” (33) while unmet need for FP was defined as “*women who were sexually active, fecund, not using any form of contraception but did not wish to become pregnant at all or within the next two years”* (34, 35).

Unmet need for FP was assessed using the questions: “*Would you like to have a child/another child, or would you prefer not to have any / any more children?*” and “*Are you or your partner currently doing something or using any method to delay or avoid getting pregnant?*” (25–31). The second question was used to assess the use of modern contraceptive use. Table 1 describes the explanatory variables included in the study and that were selected based on their availability in the datasets and their policy importance from the literature review.

**Table 1:** Operational definitions of variables.

**Variables**	**Questions and operational definitions**	**Sources**
County of residence	Nairobi, Bungoma, Kericho, Kiambu, Kilifi, Kitui, Nandi, Nyamira, Siaya, Kakamega, West Pokot	(25–31)
Age in years	Respondents were asked “How old were you on your last birthday?” and their responses categorised into 15–19, 20–34, and 35–49 years	(25–31, 36–39)
Marital status	“Are you currently married or living together with a man as if married?” (Married/cohabiting, unmarried)	(25–31, 38, 39)
Education level	“What is the highest level of school you attended?” (No formal, primary, secondary, tertiary)	(25–31, 40, 41)
Household wealth quintile	Household wealth quintiles were computed from wealth index, generated using principal component analysis of household assets and materials for the dwelling floor, roof and external wall (Poorest, poorer, middle, richer, richest)	(25–31, 42, 43)
Parity	“How many times have you given birth?” (0, 1, 2–3, 4+)	(25–31, 44, 45)
Age at sexual debut	“How old were you when you first had sexual intercourse?” (Continuous variable in years)	
Facility type	The interviewer recorded the “Type of facility” as either hospital, health centre, dispensary, and pharmacy and others.	(11, 12, 25–31)
Media exposure	“In the last 12 months have you: (a) heard about family planning on the radio? (b) seen anything about family planning on the television? (c) read about family planning in a newspaper or magazine? (d) received a voice or text message about family planning on a mobile phone? (e) Seen anything on social media (i.e., Facebook, Viber, Twitter, WhatsApp or others) about family planning?” (Yes, No)	(25–31, 46, 47)
Stockouts	“Has the (METHOD) been out of stock at any time in the last 3 months?” (Yes, No)	(11, 12, 25–31)

### Statistical Analysis

Sample characteristics were described using frequencies and percentages while factors associated with the utilisation and unmet need for modern contraceptives were assessed using bivariate and multivariable logistic regressions. For selection of variables for inclusion in the multivariable analyses, Hosmer and Lemeshow recommend that the variables to be included should be: (i) clinically important variable and (ii) variables in the univariable analyses with a *p*-value < 0.25 (48). This less stringent threshold of *p* < 0.25 helps to address the stochastic variability (univariate analysis ignores the fact that individual variables that are weakly associated with the outcome can contribute significantly when they are combined). In this study, a forward stepwise method was used to enter variables into the multivariable model to identify the model of best fit, which included all the variables based on the criteria above (48). Stata 13.0 was used for analyses, which were adjusted for the sampling design and stratification using survey weight provided in the datasets. Statistical significance was set at *p* ≤ 0.05.

## Results

### Sample Characteristics

The study included 10,474 urban women of reproductive age; a majority of who resided in Nairobi County (25.0%), were aged 20–34 years (64.5%) and had two or more children (55.4%). The mean age at sexual debut was 18 years (standard deviation: 3.2). Most women accessed FP services from health centres and dispensaries (92%) and 16.1% of health facilities reported stockouts of contraceptives within 3 months preceding the survey (Table 2).

**Table 2:** Sample characteristics of urban women of reproductive in Kenya 2014–2018.

**Variables**	**Categories**	***n* (%)**
**Sociodemographic characteristics**		
Age, years	15–19	756 (7.2)
	20–34	6,750 (64.5)
	35–49	2,968 (28.3)
Marital status	Married/cohabiting	6,847 (65.4)
	Unmarried	3,627 (35.6)
**Socioeconomic characteristics**		
Level of education	No formal	207 (2.0)
	Primary	3,950 (37.7)
	Secondary	3,780 (36.1)
	Tertiary	2,537 (24.2)
Household wealth quintile	Richest	4,202 (40.1)
	Richer	3,146 (30.0)
	Middle	1,645 (15.7)
	Poorer	829 (7.9)
	Poorest	652 (6.3)
**Reproductive health characteristics**		
Parity	None	2,029 (19.4)
	One	2,652 (25.3)
	Two to Three	3,828 (36.6)
	≥4	1,965 (18.8)
Sexual debut	Mean (SD)	18.1 (3.2)
Media exposure	No	690 (6.6)
	Yes	9,784 (93.4)
**Health systems characteristics**		
Facility Type	Hospital	459 (4.4)
	Health centre	5,695 (54.4)
	Dispensary	3,935 (37.6)
	Pharmacy and others	385 (3.7)
Stockouts	No	8,787 (83.9)
	Yes	1,687 (16.1)
Facility supports CHVs	No	3,885 (59.0)
	Yes	6,177 (37.1)
County	Nairobi	2,613 (25.0)
	Bungoma	842 (8.0)
	Kericho	1,370 (13.1)
	Kiambu	1,623 (15.5)
	Kilifi	988 (9.4)
	Kitui	798 (7.6)
	Nandi	798 (7.6)
	Nyamira	678 (6.5)
	Siaya	432 (4.1)
	Kakamega	166 (1.6)
	West Pokot	166 (1.6)
Year of survey	2014	2,556 (24.4)
	2015	2,932 (28.0)
	2016	1,690 (16.1)
	2017	1,658 (15.8)
	2018	1,638 (15.6)

*CHVs, Community health volunteers; SD, standard deviation*.

### Prevalence and Factors Associated With Modern Contraceptive Use Among Urban Women

The overall prevalence of modern contraceptive use was 53.7% (95% CI 52.1–55.3%); highest among middle-aged (57.2%) and secondary educated (54.5%) women and those from richer households (56.3%). Also, a high prevalence of modern contraceptive use was seen among women with two to three children (65.9%), who resided in Kakamega county (62.8%), were exposed to FP messages in the media (54.3%) and had access to hospitals (63.4%). The prevalence of modern contraceptive use increased from 50.3% (95% CI 46.9–53.7%) in 2014 to 55.1% (95% CI 51.8–58.3%) in 2018 (Table 3).

**Table 3:** Prevalence and factors associated with modern contraceptive use among urban women in Kenya, 2014–2018.

**Variables**	**Characteristics**	**Prevalence**		**Bivariate**			**Multivariable**		
		***n***	**Weighted %**	**cOR**	**95% CI**	***p*-value**	**aOR**	**95% CI**	***p*-value**
County	Nairobi	1,357	52.6 (49.7–55.5)	Ref			Ref		
	Bungoma	447	54.1 (50.9–57.1)	1.05	0.89–1.25	0.50	1.03	0.86–1.25	0.73
	Kericho	680	48.3 (44.3–52.3)	0.84	0.69–1.02	0.09	0.83	0.61–1.12	0.22
	Kiambu	978	57.7 (52.6–62.7)	1.23	0.97–1.56	0.09	1.30	0.93–1.81	0.12
	Kilifi	440	44.5 (40.5–48.6)	**0.72**	**0.59–0.88**	**0.002**	0.82	0.59–1.12	0.21
	Kitui	470	58.2 (53.8–62.6)	**1.25**	**1.01–1.55**	**0.04**	**1.53**	**1.04–2.24**	**0.03**
	Nandi	463	58.0 (52.2–63.7)	1.24	0.95–1.62	0.10	**1.54**	**1.02–2.33**	**0.04**
	Nyamira	358	55.0 (49.1–60.8)	1.10	0.84–1.43	0.47	1.21	0.86–1.71	0.27
	Siaya	241	56.1 (51.5–60.6)	1.15	0.92–1.43	0.20	1.15	0.86–1.55	0.34
	Kakamega	103	62.8 (53.2–71.4)	**1.51**	**1.00–2.28**	**0.05**	**1.69**	**1.16–2.45**	**0.006**
	West Pokot	73	44.8 (34.6–55.5)	0.73	0.47–1.14	0.17	0.83	0.44–1.56	0.56
Age, years	15–19	235	29.9 (25.6–34.5)	**0.41**	**0.33–0.51**	**<0.001**	**1.39**	**1.04–1.86**	**0.03**
	20–34	3,937	57.2 (55.2–59.1)	**1.30**	**1.15–1.46**	**<0.001**	**2.02**	**1.76–2.31**	**<0.001**
	35–49	1,438	50.7 (48.2–53.1)	Ref			Ref		
Marital status	Married/cohabiting	4,215	62.0 (60.3–63.6)	Ref			Ref		
	Unmarried	1,395	38.2 (35.4–41.1)	**0.38**	**0.33–0.43**	**<0.001**	**0.55**	**0.47–0.63**	**<0.001**
Parity	None	672	31.6 (28.5–34.9)	Ref			Ref		
	1	1,407	52.7 (49.5–55.9)	**2.41**	**2.05–2.83**	**<0.001**	**2.03**	**1.72–2.39**	**<0.001**
	2–3	2,452	65.9 (64.0–67.8)	**4.18**	**3.55–4.92**	**<0.001**	**4.21**	**3.46–5.12**	**<0.001**
	4+	1,079	55.1 (51.9–58.3)	**2.65**	**2.16–3.26**	**<0.001**	**3.91**	**2.96–5.17**	**<0.001**
Education	No formal	60	33.1 (24.8–42.6)	**0.42**	**0.27–0.64**	**<0.001**	**0.27**	**0.17–0.42**	**<0.001**
	Primary	2,112	53.7 (51.6–55.8)	0.99	0.86–1.14	0.93	**0.66**	**0.56–0.78**	**<0.001**
	Secondary	2,032	54.5 (51.9–57.1)	1.02	0.89–1.17	0.72	**0.85**	**0.74–0.99**	**0.04**
	Tertiary	1,406	53.9 (51.0–56.7)	Ref			Ref		
Household wealth quintiles	Richest	2,249	53.6 (51.2–56.0)	Ref			Ref		
	Richer	1,765	56.3 (53.6–59.0)	1.11	0.98–1.27	0.10	1.12	0.98–1.28	0.11
	Middle	867	52.2 (49.2–55.1)	0.94	0.80–1.10	0.47	0.95	0.78–1.14	0.57
	Poorer	433	51.2 (47.1–55.2)	0.90	0.75–1.09	0.30	0.84	0.67–1.04	0.11
	Poorest	296	45.2 (39.9–50.7)	**0.71**	**0.56–0.90**	**0.01**	**0.73**	**0.58–0.91**	**0.01**
Media exposure	No	300	43.7 (38.7–48.7)	Ref			Ref		
	Yes	5,310	54.3 (52.7–55.9)	**1.53**	**1.24–1.88**	**<0.001**	**1.30**	**1.03–1.64**	**0.02**
CHVs†	No	1,407	52.7 (49.5–55.9)	0.89	0.79–1.00	0.05	1.03	0.82–1.29	0.83
	Yes	2,452	65.9 (64.0–67.8)	Ref			Ref		
Sexual debut	Sexual debut		18.2 (3.0)	0.99	0.97–1.01	0.75	1.00	0.98–1.02	0.87
Stockouts	No	4,685	53.6 (51.8–55.3)	Ref			Ref		
	Yes	925	54.7 (51.1–58.2)	1.04	0.89–1.22	0.58	0.99	0.79–1.24	0.94
Facility type	Hospital	272	63.4 (59.1–67.6)	Ref			Ref		
	Health centre	2,891	51.5 (49.4–53.6)	**0.61**	**0.50–0.74**	**<0.001**	0.81	0.61–1.08	0.15
	Dispensary	2,252	56.6 (53.6–59.5)	**0.75**	**0.60–0.93**	**0.01**	0.77	0.58–1.02	0.07
	Pharmacy and others	195	49.9 (43.9–55.9)	**0.57**	**0.42–0.77**	**<0.001**	0.79	0.21–2.94	0.72
Survey year	2014	1,273	50.3 (46.9–53.7)	Ref			Ref		
	2015	1,627	55.1 (51.7–58.6)	1.21	0.99–1.47	0.05	**1.28**	**1.05–1.56**	**0.02**
	2016	908	54.0 (50.7–57.3)	1.16	0.95–1.40	0.13	1.21	0.98–1.48	0.08
	2017	905	55.4 (51.8–58.9)	**1.22**	**1.00–1.49**	**0.05**	**1.36**	**1.11–1.66**	**0.003**
	2018	897	55.1 (51.8–58.3)	**1.21**	**1.00–1.46**	**0.05**	**1.25**	**1.02–1.52**	**0.03**

*Ref, reference category; cOR, crude odds ratio; aOR, adjusted odds ratio; CI, confidence interval; †facility supports community health volunteers (CHVs). Bold: Statistically significant at p < 0.05*.

Modern contraceptive use was associated with the county of residence, age, marital status, parity, education, household wealth quintile, exposure to media, and survey year. The odds of modern contraceptive use were higher among teenagers (aOR 1.39, 95% CI 1.04–1.86) and middle-aged women (aOR 2.02, 95% CI 1.76–2.31) compared to women above 35 years. Also, women exposed to FP messages in the media had higher odds of modern contraceptive use (aOR 1.30, 95% CI 1.03–1.64) and those with children had a 2- to 4-fold higher odds of modern contraceptive use compared to those with no exposure or childless. Unmarried, uneducated, primary, and secondary educated women and those from poorest household had 15–73% reduced odds of modern contraceptive use. Women who resided in Kitui, Nandi and Kakamega counties had between 53 and 69% increased odds of modern contraceptive use compared to those in Nairobi (Table 3).

### Prevalence and Factors Associated With the Unmet Need of FP Among Urban Women

The overall prevalence of unmet needs of FP was 16.9% (95% CI 15.8–18.1%). It was highest among teenagers (34.6%), uneducated (30.0%), and poorest (29.5%) women and those with four or more children (24.0%), had access to health centres (18.7%) and were from Kilifi (22.7%), Kericho (22.6%), and Siaya County (21.8%) The prevalence of unmet needs of FP significantly decreased from 21.0% (95% CI 18.7–23.6%) in 2014 to 14.3% (95% CI 12.2–16.6%) in 2018 (Table 4).

**Table 4:** Prevalence and factors associated with unmet needs for FP among urban women in Kenya, 2014–2018.

**Variables**	**Characteristics**	**Prevalence**		**Bivariate**			**Multivariable**		
		***n***	**Weighted %**	**cOR**	**95% CI**	***p*-value**	**aOR**	**95% CI**	***p*-value**
County	Nairobi	370	17.1 (15.0–19.3)	Ref			Ref		
	Bungoma	135	19.3 (16.3–22.7)	1.16	0.90–1.50	0.25	0.98	0.73–1.31	0.897
	Kericho	238	22.6 (18.5–27.4)	**1.42**	**1.06–1.91**	**0.019**	1.00	0.64–1.55	0.998
	Kiambu	161	13.1 (10.7–15.9)	0.73	0.56–0.96	0.026	0.66	0.42–1.02	0.058
	Kilifi	178	22.7 (19.2–26.7)	**1.43**	**1.10–1.85**	**0.007**	1.07	0.70–1.66	0.746
	Kitui	73	11.1 (8.4–14.4)	**0.60**	**0.43–0.85**	**0.004**	**0.34**	**0.18–0.64**	**0.001**
	Nandi	120	17.5 (12.8–23.4)	1.03	0.69–1.53	0.88	0.70	0.40–1.23	0.220
	Nyamira	94	16.1 (12.1–21.2)	0.94	0.65–1.35	0.72	**0.56**	**0.32–0.98**	**0.042**
	Siaya	81	21.8 (16.5–28.2)	**1.35**	**0.94–1.97**	**0.11**	0.98	0.61–1.58	0.925
	Kakamega	23	15.8 (11.0–22.1)	0.91	0.59–1.42	0.68	0.94	0.62–1.43	0.779
	West Pokot	21	17.2 (10.6–26.6)	1.01	0.57–1.80	0.98	0.99	0.35–2.76	0.982
Age, years	15–19	186	34.6 (29.2–40.5)	2.23	1.71–2.91	**<0.001**	**2.46**	**1.68–3.62**	**<0.001**
	20–34	860	14.9 (13.7–16.1)	0.74	0.63–0.86	**<0.001**	0.85	0.70–1.04	0.110
	35–49	448	19.2 (17.1–21.5)	Ref			Ref		
Marital status	Married/cohabiting	1,051	15.9 (14.6–17.2)	Ref			Ref		
	Unmarried	443	20.6 (18.1–23.3)	**1.38**	**1.15–1.65**	**<0.001**	**1.43**	**1.20–1.70**	**<0.001**
Parity	None	286	20.5 (17.4–24.0)	Ref			Ref		
	1	301	13.9 (12.0–16.0)	0.62	0.48–0.82	**0.001**	0.77	0.59–1.01	0.063
	2–3	514	14.8 (13.4–16.4)	0.68	0.53–0.86	**0.001**	0.81	0.61–1.07	0.142
	4+	392	24.0 (21.2–27.0)	1.22	0.95–1.56	0.12	1.26	0.87–1.84	0.227
Education levels	No formal	46	30.0 (20.4–41.8)	**2.68**	**1.55–4.62**	**<0.001**	**2.77**	**1.57–4.87**	**<0.001**
	Primary	689	20.3 (18.4–22.4)	**1.59**	**1.27–2.00**	**<0.001**	**1.51**	**1.11–2.04**	**0.008**
	Secondary	492	15.3 (13.7–17.1)	1.13	0.87–1.47	0.36	1.06	0.80–1.41	0.666
	Tertiary	267	13.8 (11.6–16.4)	Ref			Ref		
Household wealth quintiles	Richest	522	14.8 (13.2–16.7)	Ref			Ref		
	Richer	414	16.6 (14.7–18.8)	1.14	0.94–1.40	0.19	1.01	0.79–1.27	0.962
	Middle	255	19.3 (16.5–22.5)	**1.37**	**1.09–1.74**	**0.008**	1.21	0.89–1.64	0.231
	Poorer	156	20.8 (17.7–24.2)	**1.50**	**1.18–1.91**	**0.001**	1.19	0.88–1.63	0.258
	Poorest	147	29.5 (24.7–34.7)	**2.40**	**1.82–3.17**	**<0.001**	**1.59**	**1.16–2.17**	**0.004**
Media exposure	No	136	21.1 (17.0–25.8)	Ref			Ref		
	Yes	1,358	16.7 (15.5–17.9)	**0.75**	**0.57–0.98**	**0.033**	0.92	0.71–1.20	0.529
CHVs†	No	604	18.8 (16.8–20.9)	1.18	1.00–1.40	0.053	1.12	0.80–1.56	0.500
	Yes	846	16.4 (15.0–17.9)	Ref			Ref		
Sexual debut	Sexual debut		17.7 (3.1)	**0.96**	**0.93–0.99**	**0.007**	1.01	0.98–1.05	0.494
Stockouts	No	1,285	17.1 (15.9–18.5)	Ref			Ref		
	Yes	209	15.3 (12.7–18.2)	0.87	0.68–1.12	0.254	1.07	0.76–1.50	0.706
Facility type	Hospital	50	12.0 (9.8–14.7)	Ref			Ref		
	Health centre	920	18.7 (17.1–20.4)	**1.68**	**1.31–2.16**	**<0.001**	**1.64**	**1.12–2.40**	**0.012**
	Dispensary	487	14.8 (13.1–16.7)	1.27	0.97–1.66	0.082	**1.86**	**1.24–2.79**	**0.003**
	Pharmacy and others	37	12.9 (8.6–19.1)	1.09	0.66–1.79	0.75	0.57	0.07–4.50	0.589
Survey year	2014	459	21.0 (18.7–23.6)	Ref			Ref		
	2015	424	15.9 (13.9–18.3)	**0.71**	**0.57–0.89**	**0.003**	**0.67**	**0.52–0.87**	**0.003**
	2016	214	15.6 (13.2–18.3)	**0.69**	**0.54–0.89**	**0.004**	**0.64**	**0.49–0.82**	**0.001**
	2017	204	14.5 (12.2–17.0)	**0.63**	**0.50–0.81**	**<0.001**	**0.61**	**0.48–0.77**	**<0.001**
	2018	193	14.3 (12.2–16.6)	**0.62**	**0.49–0.79**	**<0.001**	**0.61**	**0.47–0.78**	**<0.001**

*Ref, reference category; cOR, crude odds ratio; aOR, adjusted odds ratio; CI, confidence interval; †facility supports community health volunteers (CHVs). Bold: Statistically significant at *p* < 0.05*.

Unmet need for FP was associated with the county of residence, age, marital status, education, household wealth, parity, facility type, and year of study. The odds of unmet needs for FP among women aged 15–19 years were 2.5 times (aOR 2.46, 95% CI 1.68–3.62) higher than those of women aged 35–49 years. Women with no formal education (aOR 2.77, 95% CI 1.57–4.87) and primary education (aOR 1.51, 95% CI 1.12–2.04) had increased odds of unmet need compared to women with tertiary education. Unmarried women had 43% (aOR 1.43, 95% CI 1.19–1.70) higher odds compared to the married/cohabiting while those from the poorest households had 59% (aOR 1.59, 95% CI 1.16–2.17) higher odds of unmet need compared to from the richest households. The odds of unmet need of FP were 64% (aOR 1.64, 95% CI 1.12–2.40) and 86% (aOR 1.86, 95% CI 1.24–2.79) higher among women with access to health centres and dispensaries, respectively, compared to hospitals offering FP services. The odds of unmet need of FP were 33 and 39% lower in 2015 and 2017/2018 compared to 2014 (Table 4).

## Discussion

This study found that slightly more than half of urban women of reproductive age used modern contraceptives while about 17% of them had unmet needs for FP. Our results show that there was an increase in the prevalence of modern contraceptive use and a decrease in unmet need for FP in urban areas between 2014 and 2018, a pattern observed in other studies in Kenya (11, 12). These improved FP indicators could be attributed to the overall increase in investment in government- and partner-supported programs in urban areas (22) and the government's commitment to improving access to FP services in order to increase modern CPR to 66% in 2030 (8). However, despite the increase in modern CPR and the decrease in unmet need for FP, the rates are below the national average of 62 and 12% in 2020, respectively (9), which could indicate a possible urban-rural disparity in FP indicators and underscore the need to strengthen health systems to improve and promote equal and affordable access to FP services, especially in urban areas. Furthermore, our findings highlight a possible subnational disparity in modern contraceptive use and unmet need for FP, with West Pokot, Kilifi, and Kericho counties having the lowest modern CPR and Kilifi, Kericho, and Siaya counties having the highest unmet need of FP; findings that reinforce the resolve to promote equitable access to FP services countrywide through advocacy and strengthening of the devolved health service delivery at the county level.

Similar to previous studies (36, 37), we found an association between modern contraceptive use and women's age, with teenagers and middle-aged women having higher odds of utilisation and unmet need of FP compared to women aged above 35 years. This could be explained by the early sexual debut (18.1 years), which may result in higher demand for FP that may currently not be sufficiently addressed. This observation is further supported by a survey that found that less than half (44%) of health facilities in urban areas in Kenya offer FP services to adolescents (21) and a study in five Kenyan cities that found that 58% of health service providers imposed minimum age restrictions on some FP methods like injectables hence locking out young women (49). These findings highlight the need for continued advocacy and investment in FP in urban areas in Kenya including the establishment of youth-friendly sexual reproductive health centres to address the unmet need of FP and increase the urban modern contraceptive use.

Married urban women had a high prevalence of modern contraceptive use compared to unmarried women, which could be attributed to their high contact with health facilities during prenatal, antenatal, and postnatal care (50) and the likelihood to discuss birth spacing and limiting with their partners (51–53). However, our study also found that unmarried women had a high unmet need for FP, which confirms findings from previous studies in urban Kenya (38, 39). While some unmarried women are sexually active and desire to have children, there are likely to be teenagers or young women and not in a stable supportive relationship, which could contribute to their unmet need. Also, some health providers are known to discourage unmarried women from using contraceptives due to risk of infertility or difficulty conceiving (49).

Urban women from the poorest households and with lower education levels had higher odds of unmet need for FP and lower odds of modern contraceptive use, a finding consistent with a previous study in urban Kenya (42). With more than half of the urban population in Kenya living in informal settlements (17), poor women in these settlements are more likely to pay for contraception than their richest counterparts (43) and have poor access to healthcare including reproductive services (19) due to fewer lower-level health facilities that are majorly accessed by poor women (54). This argument is supported by our finding that women who accessed FP services from health centres and dispensaries had higher odds of unmet needs for FP compared to those with access to hospitals. In Kenya, only 73% of health facilities in urban areas offer FP services, with better availability of contraceptives in rural facilities compared to urban (21). Also, fewer health centres offer FP services compared to higher-level facilities (21) and are often understaffed, understocked and less equipped to provide some FP services (55) contributing to missed opportunities for FP services (56). On the other hand, poor women are also likely to have a low level of education despite higher education levels being positively associated with modern contraceptive use (40, 41). Educated women tend to have high levels of knowledge on reproductive health and FP (57), and increased autonomy and decision-making power on their health (58) hence their ability to decide on FP use. The persistent socioeconomic disparity in contraceptive use and unmet need for FP may erode gains made by FP programs in Kenya and hence a need to promote equity in contraceptive use in urban Kenya by ensuring equal and affordable access to contraceptive to all urban women through strengthening all healthcare levels to provide FP services.

Consistent with previous studies in urban areas (44, 45), parity was associated with higher odds of use of modern contraceptive use. Women with children are likely to use FP due to the need to space or limit birth compared to nulliparous women. However, women with four or more children also had high prevalence unmet needs for FP; a pattern observed in LMICs (47). Multiparous women have been noted to be less educated, poor (59) and with limited access to health services (60); characteristics that are associated with poor use of contraceptives (42), hence the unmet needs. They are also likely to have experienced method failure which discourages them from using FPs. Despite this, we also found that women with exposure to FP messages in the media, similar to other studies (61), had 30% higher odds of contraceptive use. Women's exposure to FP messages is likely to dispel rumours, myths and misconceptions related to contraceptives and their side effects (46, 47). This finding underscores the key role that media plays in health education, and social and behavioural change communication resulting in increased utilisation of health services including FP.

This study sought to explore factors associated with modern contraceptive use and the unmet need for FP using nationally representative data; possibly making the results generalizable to urban areas in Kenya. However, it should be noted that the study only focused on the prevalence of modern contraceptive use and not the use of any contraceptive method, which may underestimate the proportion of urban women using FP. The study also cannot infer causation due to the cross-sectional nature of the data; and some important variables like religion and user fees on FP were not included due to a higher number of missing observations. Moreover, the study defined urban areas based on the KNBS classification, as used in the dataset (25–31). While the dynamics of urban settings in Kenya are varied, the study used the official classification of urban areas which also guides government planning, development, and policies, and to allow for comparison with previous studies (25–31).

## Conclusion

Overall, slightly more than half of the urban women in Kenya used contraceptives while about one-fifth had an unmet need for FP. Over time, there was an overall increase in FP use and a simultaneous decrease in the unmet need for FP, which were associated with sociodemographic, socioeconomic, and contextual factors. Urban areas are unlikely to reach the government FP targets by 2030 if interventions to strengthen the provision of FP services will not be put in place to address the high prevalence of unmet need for FP. These interventions should be targeted towards teenagers, young, unmarried, poor, and uneducated urban women to improve uptake of FP services. Future studies on contraceptives use and unmet needs for FP should disaggregated data by area of residence to unmask possible urban-rural disparities, which needs to be explored further in Kenya.

## Data Availability Statement

Data used in this study are publicly available. This data can be found here: https://www.pma2020.org/request-access-to-datasets.

## Ethics Statement

The studies involving human participants were reviewed and approved by Kenya Medical Research Institute. Written informed consent to participate in this study was provided by the participants' legal guardian/next of kin.

## Author Contributions

All authors listed have made a substantial, direct, and intellectual contribution to the work and approved it for publication.

## Conflict of Interest

The authors declare that the research was conducted in the absence of any commercial or financial relationships that could be construed as a potential conflict of interest.

## Publisher's Note

All claims expressed in this article are solely those of the authors and do not necessarily represent those of their affiliated organizations, or those of the publisher, the editors and the reviewers. Any product that may be evaluated in this article, or claim that may be made by its manufacturer, is not guaranteed or endorsed by the publisher.

## Abbreviations

KDHS: Kenya Demographic Health Survey
CI: Confidence Interval
LMICs: Low- and middle-income countries
aOR: Adjusted odds ratio
cOR: Crude odds ratio
PMA: Performance Monitoring for Accountability
SD: Standard deviation.
